# Clinical Trauma Severity of Indoor and Outdoor Injurious Falls Requiring Emergency Medical Service Response

**DOI:** 10.21203/rs.3.rs-4202941/v1

**Published:** 2024-05-07

**Authors:** Kathryn G. Burford, Nicole G. Itzkowitz, Remle P. Crowe, Henry E. Wang, Alexander X. Lo, Andrew G. Rundle

**Affiliations:** Columbia University Mailman School of Public Health; Columbia University Mailman School of Public Health; ESO Solutions LLC; Ohio State University; Northwestern University Feinberg School of Medicine; Columbia University Mailman School of Public Health

**Keywords:** Falls, Injuries, Outdoor Falls, Surveillance

## Abstract

**Background:**

Injurious falls represent a significant public health burden. Research and polices have primarily focused on falls occurring indoors despite evidence that outdoor falls account for 47–58% of all falls requiring some medical attention. This study compared the clinical trauma severity of indoor versus outdoor injurious falls requiring Emergency Medical Services (EMS) response.

**Methods:**

Using the 2019 National Emergency Medical Services Information System (NEMSIS) dataset, we identified the location of patients injured from falls that required EMS response. We classified injury severity using 1) the Revised Trauma Score for Triage (T-RTS): ≤ 11 indicated the need for transport to a Trauma Center; 2) Glasgow Coma Scale (GCS): ≤8 and 9–12 indicated moderate and severe neurologic injury; and 3) patient clinical acuity by EMS: Dead, Critical, Emergent, Low.

**Results:**

Of 1,854,909 encounters for patients with injurious falls, the vast majority occurred indoors (*n*=1,596,860) compared to outdoors (*n*=152,994). The proportions of patients with moderate or severe GCS scores, were comparable between those with indoor falls (3.0%) and with outdoor falls on streets or sidewalks (3.8%), T-RTS scores indicating need for transport to a Trauma Center (5.2% vs 5.9%) and EMS acuity rated as Emergent or Critical (27.7% vs 27.1%).Injurious falls were more severe among male patients compared to females: and males injured by falling on streets or sidewalks had higher percentages for moderate or severe GCS scores (4.8% vs 3.6%) and T-RTS scores indicating the need for transport to a Trauma Center (7.3% vs 6.5%) compared to indoor falls. Young and middle-aged patients whose injurious falls occurred on streets or sidewalks were more likely to have a T-RTS score indicating the need for Trauma Center care compared to indoor falls among this subgroup. Yet older patients injured by falling indoors were more likely to have a T-RTS score indicating the need for Trauma Center than older patients who fell on streets or sidewalks.

**Conclusions:**

There was a similar proportion of patients with severe injurious falls that occurred indoors and on streets or sidewalks. These findings suggest the need to determine outdoor environmental risks for outdoor falls to support location-specific interventions.

## Background

Falls represent an enormous global public health burden associated with significant disability and mortality, with a worldwide age-standardized incidence of 2,238 falls per 100,000 persons per year in 2017, over 16.6 million years of life lost, and an average loss of 4% of one’s full health status from one fall (James et al., 2020). For the U.S., the Centers for Disease Control (CDC) reported 7.9 million unintentional injurious falls in 2019 that was associated with 131.5 billion USD in medical costs (WISQARS (Web-Based Injury Statistics Query and Reporting System) | Injury Center | CDC, 2023). Although falls affect all ages, the burden of falls in the U.S. is disproportionately borne by older persons, for whom falls are the leading cause of disability and functional decline (James et al., 2020; Panel on Prevention of Falls in Older Persons, American Geriatrics Society and British Geriatrics Society, 2011).

Research and policy attention has been primarily devoted to falls occurring indoors,(“Guideline for the Prevention of Falls in Older Persons. American Geriatrics Society, British Geriatrics Society, and American Academy of Orthopaedic Surgeons Panel on Falls Prevention,” 2001; Panel on Prevention of Falls in Older Persons, American Geriatrics Society and British Geriatrics Society, 2011) despite reports of outdoor falls accounting for 47–58% of all falls among community-dwelling adults (Li et al., 2006; Timsina et al., 2017). Risk factors for indoor and outdoor falls also tend to differ. Indoor falls are more likely to be influenced by personal risk factors, the indoor environment, and underlying health conditions (Schepers et al., 2017). Outdoor falls occur among more active individuals and are primarily influenced by outdoor environmental factors (Schepers et al., 2017). These associated outdoor environmental factors have included weather conditions (e.g., snow and ice or extreme heat) and the physical environment (e.g., uneven surfaces, slippery surfaces, stairs) (Timsina et al., 2017). However there remains limited understanding for the role of urban design and the environment on the risk of outdoors falls, especially for falls on streets and sidewalks, where 85% of outdoor falls requiring an Emergency Medical Services (EMS) response were found to occur (Rundle et al., 2023, 2024). Additionally, there is an abundance of literature addressing the risk factors for falls related to the older population, but even for this demographic subgroup, research studies for outdoor falls were far fewer (Chippendale et al., 2023), with substantially greater focus on person-level and indoor environment characteristics (e.g. home trip hazards) (“Guideline for the Prevention of Falls in Older Persons. American Geriatrics Society, British Geriatrics Society, and American Academy of Orthopaedic Surgeons Panel on Falls Prevention,” 2001; Montero-Odasso et al., 2022; Panel on Prevention of Falls in Older Persons, American Geriatrics Society and British Geriatrics Society, 2011). These major knowledge gaps have hampered the development of community-specific and person-centered prevention strategies to reduce the burden of outdoor injurious falls (Chippendale et al., 2017; Li et al., 2006).

A primary reason for the lack of evidence to determine environmental risk factors for outdoor falls, and particularly at the population-level, is limitations in the surveillance of falls. The three primary public health surveillance systems in the U.S. for fall-related injuries are the CDC’s Web-Based Injury Statistics Query and Reporting System (WISQARS), the Behavioral Risk Factor Surveillance System (BRFSS) and the National Health Interview Survey (NHIS) and yet none of these systems routinely provide data on the locations in which falls occur (Moreland et al., 2020; Timsina et al., 2017; *WISQARS (*Web-Based Injury Statistics Query and Reporting System) | Injury Center | CDC, 2023). While the NHIS has been used to examine outdoor falls, these analyses are 10–25 years old, likely because the location of the fall is determined by intensive coding of unstructured narrative texts, which makes use of these data cumbersome to analyze (Timsina et al., 2017). To improve the surveillance of outdoor falls, Rundle et al. (2023) developed a methodology to identify injurious falls by indoor versus outdoor location using EMS clinical and administrative data from the National Emergency Medical Services Information System (NEMSIS) (Rundle et al., 2023). Among the 1,854,909 injuries from falls that required an EMS response in 2019, 129,408 of these fall injuries were identified as occurring outdoors on streets and sidewalks, a number which is 70% higher than the number of pedestrians reportedly injured by automobiles (Li et al., 2006; National Highway Traffic Safety Administration, n.d.; Timsina et al., 2017). While establishing this surveillance method was a critical first step towards developing interventions to reduce outdoor falls, epidemiological data are needed to improve understanding of the public health and clinical burden of outdoor falls.

There are very few available studies to compare the severity of indoor and outdoor falls, with the focus of these being among the older adult population (Bath & Morgan, 1999; Chippendale et al., 2017; Kelsey et al., 2010, 2012; Kim, 2016; Lee, 2021; O’Loughlin et al., 1994). Chippendale et al.’s (2016) study of older U.S. trauma center patients who sustained outdoor falls had a higher frequency of open wounds and head injuries compared to indoor falls, but no significant difference on the injury severity score (ISS) (Chippendale et al., 2017). Kim (2016) found that outdoor falls led to a higher proportion of head and neck injuries than indoor falls among older emergency department patients across 20 hospitals in Korea (Kim, 2016). Jung et al. (2017) determined that the likelihood of severe injury, as determined by level of care, from outdoor falls in older adults was higher in men compared to women (Jung et al., 2018). These studies were limited by examining only individuals admitted to a hospital, and none were of a nationally representative sample. Furthermore, assessment of outcomes and severity of falls varied across these studies. Improved understanding of the clinical severity for fall injuries is critical to determining the short- and long-term burdens of 1) morbidities and disability among individuals (Stewart Williams et al., 2015) and 2) health care utilization needs of different communities (Eliacin et al., 2021; Korley et al., 2016), particularly in light of potential differences across sociodemographic groups (Chun Fat et al., 2019; Sharma et al., 2018). In the immediate setting following an injury, clinical severity scoring tools have been recommended, including the Revised Trauma Score for Triage (T-RTS) and Glasgow Coma Scale (GCS), to help guide on-scene EMS to determine the severity of the injury and optimal care response for the individual (Champion et al., 1989; Newgard et al., 2022). However, there are no population-level studies that have characterized the distribution of such scores in the assessment of severity of indoor versus outdoor injurious falls.

National surveillance data comparing indoor and outdoor injurious falls are critical to the development of person-centered, community-specific programs and policies to prevent serious falls. Data from EMS responses on the severity and level of care for indoor and outdoor injurious falls could be particularly informative from a healthcare resource perspective. Here we use 2019 national U.S. EMS data to compare the clinical trauma severity of indoor versus outdoor injurious falls, and further to examine these patterns by patient demographic characteristics.

## Methods

### Study Design

This cross-sectional study of EMS records from the 2019 National Emergency Medical Services Information System (NEMSIS) Public-Release Research Dataset included 1,854,909 occurrences of falls requiring EMS response across US states and territories (Rundle et al., 2023).

### Data Source

NEMSIS is the national system to collect and standardize data from EMS agencies across the US. The National Highway Traffic Safety Administration Office for Emergency Medical Services provides these NEMSIS data are public release, de-identified, and HIPAA exempt data released by the University of Utah, as such further Institutional Review Board (IRB) review was not requested (Dawson, 2006; Ehlers et al., 2023). The use of NEMSIS to identify falls and locations of falls have been previously described and includes a robust approach to identifying overall injurious falls and to identifying falls for which syncope (heat-related and non-heat related syncope) and heat illness were contributing factors. (Rundle et al., 2023). EMS data entry into NEMSIS must abide by the standards set forth by the NHTSA Office of EMS and outlined in the NEMSIS data dictionary (https://nemsis.org/media/nemsis_v3/release-3.5.0/DataDictionary/PDFHTML/EMSDEMSTATE/index.html) (V3 Data Dictionaries & XSD, n.d.).

### Study Variables and Inclusion Criteria

Detailed methods for inclusion criteria and coding of fall locations (indoor, outdoor – not on street or sidewalk, outdoor – on street or sidewalk, indoor/outdoor unclear), patient demographic variables, and on-scene clinical measures (e.g. patient acuity) can be found in Rundle et al (Rundle et al., 2023). Briefly, we used NEMSIS variables ePatient 13, ePatient 15 and eSituation 13 to define patient sex (male, female), and age groups (0–20, 21–30, 31–40, 41–50, 51–60, 61–65, 66–70, 71–75, 76–80, 81–85, 86–90 and 91 + years), respectively. Falls with EMS notations of seizures have been removed from the analyses. NEMSIS data includes the patient’s clinical acuity, rated by EMS, which is classified as: Dead Without Resuscitation Efforts, Critical, Emergent, Low, Unknown. NEMSIS data were also used to calculate the Revised Trauma Score for Triage (T-RTS) and the Glasgow Coma Scale (GCS). The T-RTS, GCS and patient acuity data were used to characterize the severity of the injuries as observed by the EMS clinician on scene. When the tests used to calculate the GCS and the T-RTS were administered multiple times for a patient, the mean of all administrations were used.

#### Revised Trauma Score for Triage (T-RTS)

The Revised Trauma score for Triage to a Trauma Center (T-RTS) is a modified version of the original Trauma Score, that is more reliable and excludes capillary refill and respiratory expansion which are more difficult to assess in the field by EMS (Champion et al., 1989). The T-RTS can be used by EMS to make decisions on trauma care based on the severity of patient injuries (Lichtveld et al., 2008). For each patient, we calculated the mean T-RTS by summing the average value for GCS, systolic blood pressure, and respiratory rate. Champion and colleagues evaluated T-RTS cut-points based upon survival probabilities to create decision rules for patients to be triaged to a trauma center (Champion et al., 1989). Decision Rule 2 was used for the present study to categorize the T-RTS score into: ≤11, indicated need for immediate transport to a Trauma Center designated hospital; >11 does not. As blood pressure may be artificially lowered by anti-hypertensive medications, the use of which differs by age group, the validity of the T-RTS may vary by age. Therefore, assessment of the GCS alone offers a second measure of injury severity that is independent of blood pressure.

#### Glasgow Coma Scale (GCS)

The Glasgow Coma Scale (GCS) is a well-established measure of neurological status used for risk stratification in acute neurosurgical or traumatic injuries; the scale ranges from 3 to 15 and is calculated by summing the values for eye opening, verbal response, and motor response for each patient.^11^ In contemporary clinical practice, the GCS is used to determine the risks of mortality and morbidity, and more urgently to guide acute clinical management. We calculated the mean Glasgow Coma Scale (GCS) by summing the average value for eye opening, verbal response, and motor response for each patient. GCS ranges from 3 to 15, and for this analysis, GCS we use the widely used classifications for injury severity: as severe, ≤ 8; moderate, 9–12; and minor, ≥ 13 (Jain & Iverson, 2024).

### Statistical Analyses

We conducted descriptive analyses for all EMS encounters for injurious falls by comparing the T-RTS, GCS, and patient acuity classifications by fall location, and further described these analyses by patient demographics. Due to the large size of this dataset, we did not include measures of statistical significance and instead allow the readers to make interpretations based on practical relevance in the differences. We conducted all analyses in R statistical software (v4.3.1; R Core Team 2023).

## Results

In total 1,854,909 injuries from falls that required an EMS response were identified in the 2019 NEMSIS data. As reported previously (Rundle et al., 2023) and here in Table 1, of these falls the majority, 1,596,860, occurred indoors, 152,994 falls occurred outdoors, of which 129,408 occurred on streets and sidewalks. For 53,700 falls, the indoor versus outdoor status of the fall location could not be ascertained from the coded data, and for 51,355 the location data were not reported. Overall, 28% of fall patients injured by a fall had an EMS acuity rating of Critical or Emergent and 0.1% Dead at Scene (see Table 1). There were a minority of injured fall patients (n = 51,422; 3.0%) who had moderate or severe GCS scores and a minority of patients (n = 84,220; 5.2%) who had a T-RTS score indicating the need for transport to a Trauma Center. The proportion of patients injured by falling indoors who had an Emergent or Critical acuity rating was similar to the proportion of patients injured by falls occurring outdoors on streets or sidewalks (27.7% vs. 27.1%). Similarly, the proportion of patients injured by falling indoors and having moderate or severe GCS scores was comparable to the proportion of patients with moderate or severe GCS scores injured by falling on a street or sidewalk (3.0% vs. 3.8%). Likewise, the proportion of injured patients who fell indoors and who had a T-RTS score indicating the need for transport to a Trauma Center was comparable to the proportion of injured patients who had a T-RTS score indicating the need for transport to a Trauma Center and fell outdoors on a street or sidewalk (5.2% vs 5.9%). Patients injured by falling outdoors not on a street or sidewalk had a lower percentage for moderate or severe GCS scores (2.2% vs. 3.8%) and for T-RTS scores indicating the need for transport to a Trauma Center (4.4% vs 5.9%) compared to percentages for those injured by falling outdoors on streets or sidewalks, but the reverse pattern was true for patients injured by falls with an Emergent or Critical patient acuity (31.1% vs 27.1%).

Male patients injured by falls were more likely to have an acuity rated as Critical or Emergent (29.4% vs. 26.4%), moderate to severe GCS scores (3.7% vs. 2.3%) and have a T-RTS score indicating the need for transport to a Trauma Center (6.5% vs. 4.3%) than female patients injured by falls (see Table 2). The proportions for male patients injured by falls who had moderate or severe GCS scores (4.8% vs 3.6%) and T-RTS scores indicating the need for transport to a Trauma Center (7.3% vs 6.5%) were higher for outdoor falls on streets or sidewalks compared to indoor falls. While among female patients injured by falls, the percentages for moderate or severe GCS scores (1.8% vs 2.2%) and T-RTS scores necessitating care at a Trauma Center (3.9% vs 4.3%) were similar for falls on streets or sidewalks and indoors locations. The proportions for injurious falls for which patient acuity was rated Critical or Emergent were similar for falls occurring on streets or sidewalks and for falls occurring indoors among both male (28.2% vs 29.4%) and female patients (25.4% vs 26.5%).

Tables 3–5 report on the injury severity measures by location of fall and by patient age. Young and middle-aged patients (≤ 60 years) who were injured by falls on streets and sidewalks were more likely to have a T-RTS score indicating the need for transport to a Trauma Center compared to young and middle-aged patients injured by falls occurring indoors. However, older patients (> 60 years) who were injured by falls indoors were more likely to have a T-RTS score indicating the need for transport to a Trauma Center than older adults who fell on streets or sidewalks. Similar patterns were observed for patients injured by falls who had moderate or severe GCS scores.

## Discussion

The majority of fall injuries to which EMS responded in 2019 occurred indoors, with the second largest category occurring outdoors on streets or sidewalks. However, the proportions for patients who had fall injuries rated as Emergent or Critical, having moderate or severe GCS scores and having a T-RTS score indicating the need for transport to a Trauma Center were similar across indoor and outdoor locations of falls. Given the large numbers of falls that occur indoors and among older persons, it is appropriate that falls prevention guidelines and recommendations have focused on these falls (“Guideline for the Prevention of Falls in Older Persons. American Geriatrics Society, British Geriatrics Society, and American Academy of Orthopaedic Surgeons Panel on Falls Prevention,” 2001; Panel on Prevention of Falls in Older Persons, American Geriatrics Society and British Geriatrics Society, 2011). However, the comparable trauma severity of outdoor injurious falls to those that occur indoors and the greater severity of falls on streets and sidewalks among young and middle-aged patients suggests that additional public health attention is needed to identify modifiable outdoor environmental risk factors to prevent outdoor falls.

This study found that the proportion of severe outdoor falls on streets and sidewalks was higher among men compared to women. This finding may be explained by differences in the age and physical activity status for men and women falling outside versus inside (Duckham et al., 2013; Kelsey et al., 2012; Timsina et al., 2017). Timsina and colleagues (2017) found that young and middle-age men were more likely to fall outside, whereas older females are were more likely to fall inside (Timsina et al., 2017). Young men were also most likely to fall while engaging in vigorous activity, and thus the potentially higher speed and impact of the activities at the time of falling may result in more serious fall injuries for this subgroup. An additional explanation is that men of all ages tend to consume more alcohol than women, and acute alcohol consumption is associated with greater risk of injurious falls by impacting balance control and cognition (Taylor et al., 2010). One study found that alcohol-related fall injury presentations to emergency departments (ED) were more prevalent among men and younger patients, and were more severe based on triage scale ratings and admissions to the ED compared to non-alcohol-related injuries (Woods et al., 2019). An additional study of outdoor falls similarly found that the proportion of men and the rate of alcohol consumption were higher in severe outdoor falls compared to non-severe falls (Jung et al., 2018). Future work in this area should examine the role of alcohol in the severity of injurious falls by location to support place-based intervention strategies and policies. Falls that occur on streets and sidewalks that involve alcohol may be in close proximity to businesses that serve alcohol, which is consistent with a higher density of alcohol serving establishments being linked to increased alcohol consumption and increased pedestrian injury from motor vehicles (Gruenewald et al., 1993). However, the association between injurious falls on streets and sidewalks and proximity to alcohol serving establishment has yet to be examined and is an area ripe for future research (DiMaggio et al., 2016; LaScala et al., 2001; Lasota et al., 2020; Sebert Kuhlmann et al., 2009; Treno et al., 2007; White, 2020).

We also found that outdoor falls on streets or sidewalks were more likely to result in severe injury among young and middle-aged individuals compared to indoor falls among this age subgroup, but this pattern was reversed for older adults. This finding may also be explained by the evidence showing a higher proportion of alcohol involved falls among younger adults and greater severity of these falls, which may be occurring on streets or sidewalks near alcohol serving establishments or nightlife districts (Woods et al., 2019). Indoor falls may appear to be more frequently severe among older adults due to a form of selection bias. The population of older adults that fall indoors may be more likely to be frail, while those who fall while outdoors may be in better overall health (Kelsey et al., 2012). This is consistent with findings that suggest that outdoor falls are experienced more by healthy and activity individuals, compared to greater risk of falling indoors for individuals in poorer health who may experience worse injury outcomes. (Kelsey et al., 2010; Li et al., 2006). There may also be differences in the types of surfaces (e.g., wooden floor, grass) or floor characteristics adults are falling on indoors compared to outdoors which could influence injury severity; however, studies are lacking in this area of research (Jung et al., 2018).

This analysis suggests that outdoor falls, particularly on streets and sidewalks, present a significant medical and public health burden. Current fall prevention guidelines did not explicitly examine the impact of outdoor environments falls (Montero-Odasso et al., 2022; Panel on Prevention of Falls in Older Persons, American Geriatrics Society and British Geriatrics Society, 2011) and pedestrian safety policies are largely centered around pedestrian injuries from motor vehicles with minimal attention to outdoor falls, even though these two injury types occur in the same or adjacent physical environments (Evenson et al., 2018). This may be due to the limited empirical evidence available to determine modifiable environmental risk factors for outdoor falls (Schepers et al., 2017). Li et al. (2006) found that among a sample of U.S. adults, participants subjectively reported that most (73%) of outdoor falls were due to environmental factors such as the condition of the walking surface, and usually occurred on sidewalks, curbs and streets (Li et al., 2006).Yet, rigorous epidemiological studies are needed to identify potential environmental hazards on sidewalks and streets such as street trees that may cause buckling or damage to sidewalks and increase fall risk (Bentley, 1998; Bentley & Haslam, 2001; David & Freedman, 1990; Fothergill et al., 1995; Hunt et al., 1991). This evidence would inform policy interventions to prevent outdoor fall injuries that we show are comparably severe to indoor fall injuries.

The primary strength of this study is the use of NEMSIS data, which provides a well-documented national census of health encounters requiring an EMS response. The data include pertinent sociodemographic and clinical information, and variables that can be used to code the location of the encounter, eliminating the need to incorporate additional data sources. The severity outcomes used in this analysis relied on data recorded in the EMS notes with varying degrees of missingness. We were unable to calculate GCS for 7.5% of patients and T-RTS for 13.0% of patients, and 25.9% of patients did not have an acuity measure reported. This study is also limited by missing data on fall location. The coding schema used to classify fall location does not make use of narratives and text notes created by EMS personnel, and therefore may result in misclassification of fall location. Machine learning for natural language processing applied to EMS narrative notes could supplement the ICD 10-based case-finding algorithm used in this study and increase the sensitivity of identifying fall location from EMS data (Mayampurath et al., 2021; Zhao et al., 2021). Lastly, the quality of data provided to NEMSIS may vary by region, EMS service and third-party data management systems. Combined these limitations may have caused underestimates of the numbers of falls occurring outdoors and the severity of falls by location.

## Conclusion

In conclusion, these data show that there is a significant clinical burden associated with outdoor falls, and that the proportion of severe life-threatening injuries from falls that occur outdoors is comparably substantial to that for falls that occur indoors. These findings represent a major public concern as the aging population projects an expected growth of persons 65 years and older by 22% by 2040, and the number of injurious falls and associated healthcare costs will simultaneously increase (2021 Profile of Older Americans, 2022). Indeed, recent data already shows this rising incidence of falls of 1.5% per year from 2016–2019 (Hoffman et al., 2022). These concerns emphasize the need to address outdoor falls in current fall prevention guidelines, and to improve surveillance tools for monitoring outdoor falls and associated risk factors and outcomes.

## Figures and Tables

**Table 1:** Descriptive statistics for EMS Encounters for Reported Location of Fall Injuries by Patient Acuity, GCS and T-RTS scores.^1^

	Overall		Indoor		Outdoor- Street or Sidewalk		Outdoor- Not on Street or Sidewalk		Indoor/outdoor unclear		Missing	
	N = 1854909		N = 1596860		N = 129408		N = 23586		N = 53700		N = 51355	
**Patient Acuity**												
*Dead*	1600	0.1%	1432	0.1%	92	0.1%	29	0.2%	32	0.1%	15	0.1%
*Critical*	40609	3.0%	35267	2.9%	3044	3.2%	890	4.8%	1073	4.2%	335	2.1%
*Emergent*	339764	24.8%	302119	24.8%	22522	23.9%	4840	26.3%	6955	27.2%	3328	21.0%
*Low*	991674	72.3%	880467	72.3%	68834	72.9%	12681	68.9%	17498	68.5%	12194	76.9%
*Unknown*	481262	25.9%	377575	23.6%	34916	27.0%	5146	21.8%	28142	52.4%	35483	69.1%
**GCS**												
*Severe*	16508	1.0%	13753	0.9%	1614	1.4%	214	1.0%	613	1.2%	314	0.8%
*Moderate*	34914	2.0%	30227	2.0%	2922	2.5%	257	1.2%	848	1.7%	660	1.6%
*Minor*	1663581	97.0%	1439013	97.0%	114490	96.2%	20843	97.8%	49624	97.1%	39611	97.6%
*Unknown*	139906	7.5%	113867	7.1%	10382	8.0%	2272	9.6%	2615	4.9%	10770	21.0%
**T-RTS**												
*Need for transport to Trauma Center*	84220	5.2%	72748	5.2%	6494	5.9%	849	4.4%	2408	5.0%	1721	4.5%
*Does not*	1528682	94.8%	1324148	94.8%	104366	94.1%	18250	95.6%	45534	95.0%	36384	95.5%
*Unknown*	242007	13.0%	199964	12.5%	18548	14.3%	4487	19.0%	5758	10.7%	13250	25.8%

1 GCS, Glasgow Comma Scale; T-RTS, Revised Trauma Score for Triage

**Table 2:** Descriptive Statistics for EMS Encounters for Reported Location of Fall Injuries by Patient Acuity, GCS and T-RTS Scores, Categorized by Sex.^1^

	Overall		Indoor		Outdoor- Street or Sidewalk		Outdoor- Not on Street or Sidewalk		Indoor/outdoor unclear		Missing	
	N = 1854909		N = 1596860		N = 129408		N = 23586		N = 53700		N = 51355	
**Acuity**												
Male												
*Dead*	983	0.2%	868	0.2%	56	0.1%	27	0.3%	26	0.2%	6	0.2%
*Critical*	21729	3.8%	18137	3.7%	2098	3.8%	572	5.9%	727	5.5%	195	3.8%
*Emergent*	147941	25.6%	126701	25.7%	13331	24.4%	2703	27.9%	3762	28.6%	1444	25.6%
*Low*	406624	70.4%	347588	70.5%	39171	71.7%	6402	66.0%	8626	65.6%	4837	70.4%
*Unknown*	202742	26.0%	151433	23.5%	20051	26.8%	2709	21.8%	13809	51.2%	14740	69.5%
Female												
*Dead*	559	0.1%	36	0.1%	2	0.1%	6	0.0%	8	0.0%	611	0.0%
*Critical*	16981	2.4%	930	2.3%	311	2.4%	340	3.6%	138	2.8%	18700	1.5%
*Emergent*	174488	24.1%	9105	24.1%	2115	23.0%	3153	24.4%	1830	25.6%	190691	20.0%
*Low*	530657	73.5%	29465	73.4%	6244	74.5%	8799	72.0%	7160	71.5%	582325	78.4%
*Unknown*	223437	25.8%	14541	23.6%	2395	26.9%	14227	21.6%	20503	53.6%	275103	69.2%
Missing Sex	7460	0.4%	6011	0.4%	624	0.5%	106	0.4%	225	0.4%	494	1.0%
**GCS**												
Female												
*Severe*	5916	0.6%	5295	0.6%	301	0.6%	48	0.4%	160	0.6%	112	0.4%
*Moderate*	16757	1.7%	15398	1.6%	659	1.2%	75	0.7%	281	1.1%	344	1.2%
*Mild*	966752	97.7%	860083	90.9%	48921	90.5%	9908	89.5%	24860	93.7%	22980	77.5%
*Missing*	78005	7.3%	65346	6.9%	4196	7.8%	1036	9.4%	1224	4.6%	6203	20.9%
Male												
*Severe*	10540	1.4%	8416	1.3%	1309	1.8%	165	1.3%	451	1.7%	199	0.9%
*Moderate*	18041	2.3%	14732	2.3%	2250	3.0%	182	1.5%	565	2.1%	312	1.5%
*Mild*	691717	88.7%	574772	89.1%	65211	87.3%	10862	87.5%	24697	91.3%	16275	76.7%
*Missing*	59721	7.7%	46807	7.3%	5937	7.9%	1204	9.7%	1337	4.9%	4436	20.9%
Missing Sex	7460	0.4%	6011	0.4%	624	0.5%	106	0.4%	225	0.4%	494	1.0%
**T-RTS**												
Male												
*Need for transport to Trauma Center*	43,823	6.5%	36,305	6.5%	4,659	7.3%	539	5.4%	1,471	6.1%	849	5.4%
*Does not*	628,624	93.5%	522,887	93.5%	59,069	92.7%	9,444	94.6%	22,451	93.9%	14,773	94.6%
*Missing*	107,572	13.8%	85,535	13.3%	10,979	14.7%	2,430	19.6%	3,028	11.2%	5,600	26.4%
Female												
*Need for transport to Trauma Center*	40132	4.3%	36222	4.3%	1811	3.9%	310	3.4%	929	3.9%	860	3.9%
*Does not*	895796	95.7%	797768	95.7%	45008	96.1%	8748	96.6%	22944	96.1%	21328	96.1%
*Missing*	131502	12.3%	112132	11.9%	7258	13.4%	2009	18.2%	2652	10.0%	7451	25.1%
Missing Sex	7460	0.4%	6011	0.4%	624	0.5%	106	0.4%	225	0.4%	494	1.0%

1 GCS, Glasgow Comma Scale; T-RTS, Revised Trauma Score for Triage

**Table 3:** Descriptive Statistics for EMS Encounters for Reported Location of Fall Injuries by GCS, Categorized by Age.1

	Overall		Indoor		Outdoor- Street or Sidewalk		Outdoor- Not on Street or Sidewalk		Indoor/outdoor unclear		Missing	
	N = 1854909		N = 1596860		N = 129408		N = 23586		N = 53700		N = 51355	
**0–20**												
*Severe*	809	0.8%	572	0.7%	133	1.5%	29	0.5%	64	0.7%	11	0.5%
*Moderate*	1602	1.5%	1234	1.5%	221	2.5%	48	0.8%	77	0.9%	22	0.9%
*Mild*	104081	97.7%	78333	97.7%	8584	96.0%	6252	98.8%	8584	98.4%	2328	98.6%
*Missing*	10713	9.1%	7963	9.0%	919	9.3%	692	9.9%	629	6.7%	510	17.8%
**22–30**												
*Severe*	894	1.5%	563	1.3%	242	2.3%	21	1.3%	55	1.4%	13	1.0%
*Moderate*	1483	2.5%	974	2.3%	362	3.4%	38	2.3%	86	2.2%	23	1.7%
*Mild*	56894	96.0%	40338	96.3%	9915	94.3%	1620	96.5%	3731	96.4%	1290	97.3%
*Missing*	4811	7.5%	3216	7.1%	936	8.2%	188	10.1%	218	5.3%	253	16.0%
**31–40**												
*Severe*	1205	1.6%	829	1.5%	255	2.1%	26	1.4%	73	1.7%	22	1.4%
*Moderate*	1994	2.6%	1349	2.4%	495	4.0%	31	1.7%	88	2.1%	31	2.0%
*Mild*	72806	95.8%	53734	96.1%	11678	94.0%	1805	96.9%	4053	96.2%	1536	96.7%
*Missing*	6004	7.3%	4198	7.0%	1104	8.2%	181	8.9%	213	4.8%	308	16.2%
**41–50**												
*Severe*	1404	1.5%	1037	1.4%	236	1.7%	35	1.9%	75	1.7%	21	1.1%
*Moderate*	2334	2.4%	1690	2.3%	472	3.5%	20	1.1%	116	2.6%	36	1.8%
*Mild*	93031	96.1%	72137	96.4%	12839	94.8%	1807	97.0%	4343	95.8%	1905	97.1%
*Missing*	7444	7.1%	5452	6.8%	1174	8.0%	195	9.5%	203	4.3%	420	17.6%
**51–60**												
*Severe*	2271	1.2%	1851	1.2%	262	1.2%	31	1.2%	89	1.2%	38	1.0%
*Moderate*	3990	2.1%	3086	2.1%	639	2.9%	43	1.7%	150	2.1%	72	1.9%
*Mild*	180072	96.6%	145551	96.7%	21454	96.0%	2477	97.1%	6935	96.7%	3655	97.1%
*Missing*	14168	7.1%	10975	6.8%	1767	7.3%	311	10.9%	301	4.0%	814	17.8%
**61–65**												
*Severe*	1417	1.1%	1203	1.1%	113	1.0%	13	0.9%	61	1.4%	27	1.0%
*Moderate*	2384	1.9%	2016	1.9%	243	2.1%	17	1.1%	72	1.7%	36	1.4%
*Mild*	124861	97.0%	105365	97.0%	11367	97.0%	1487	98.0%	4100	96.9%	2542	97.6%
*Missing*	9724	7.0%	7922	6.8%	924	7.3%	146	8.8%	173	3.9%	559	17.7%
**66–70**												
*Severe*	1497	1.0%	1304	1.0%	99	1.0%	17	1.2%	54	1.4%	23	0.8%
*Moderate*	2553	1.8%	2320	1.8%	123	1.3%	14	1.0%	44	1.1%	52	1.8%
*Mild*	140898	97.2%	123198	97.1%	9534	97.7%	1420	97.9%	3860	97.5%	2886	97.5%
*Missing*	11168	7.2%	9360	6.9%	808	7.6%	146	9.1%	183	4.4%	671	18.5%
**71–75**												
*Severe*	1493	0.9%	1337	0.9%	84	1.0%	16	1.2%	38	1.0%	18	0.5%
*Moderate*	3062	1.8%	2856	1.9%	99	1.1%	10	0.7%	46	1.2%	51	1.4%
*Mild*	163497	97.3%	146552	97.2%	8464	97.9%	1351	98.1%	3610	97.7%	3520	98.1%
*Missing*	13134	7.2%	11195	6.9%	689	7.4%	104	7.0%	154	4.0%	992	21.7%
**76–80**												
*Severe*	1578	0.9%	1451	0.9%	51	0.7%	13	1.2%	27	0.8%	36	0.8%
*Moderate*	3471	1.9%	3257	2.0%	91	1.2%	7	0.6%	47	1.4%	69	1.6%
*Mild*	178017	97.2%	162117	97.2%	7397	98.1%	1072	98.2%	3233	97.8%	4198	97.6%
*Missing*	14677	7.4%	12491	7.0%	647	7.9%	107	8.9%	175	5.0%	1257	22.6%
**81–85**												
*Severe*	1386	0.7%	1273	0.7%	46	0.7%	3	0.4%	28	0.9%	36	0.7%
*Moderate*	3889	2.0%	3725	2.0%	37	0.6%	9	1.2%	40	1.3%	78	1.5%
*Mild*	192234	97.3%	177428	97.3%	6189	98.7%	741	98.4%	2956	97.8%	4920	97.7%
*Missing*	15326	7.2%	13146	6.7%	521	7.7%	68	8.3%	134	4.2%	1457	22.4%
**86–90**												
*Severe*	1281	0.7%	1211	0.7%	26	0.6%	3	0.7%	18	0.8%	23	0.4%
*Moderate*	3845	2.0%	3677	2.1%	45	1.1%	9	2.0%	39	1.7%	75	1.4%
*Mild*	185542	97.3%	173300	97.3%	4209	98.3%	446	97.4%	2299	97.6%	5288	98.2%
*Missing*	15636	7.6%	13391	7.0%	342	7.4%	59	11.4%	110	4.5%	1734	24.4%
**91+**												
*Severe*	1034	0.6%	951	0.6%	25	1.1%	4	1.6%	18	1.0%	36	0.7%
*Moderate*	3890	2.3%	3714	2.4%	39	1.7%	5	1.9%	31	1.8%	101	1.9%
*Mild*	162219	97.1%	152813	97.0%	2242	97.2%	248	96.5%	1677	97.2%	5239	97.5%
*Missing*	13867	7.7%	11931	7.0%	187	7.5%	33	11.4%	56	3.1%	1660	23.6%
Missing Age	13319	0.7%	11274	0.7%	1080	0.8%	168	0.7%	334	0.6%	463	0.9%

1 GCS, Glasgow Comma Scale

**Table 4:** Descriptive Statistics for EMS Encounters for Reported Location of Fall Injuries by Patient Acuity, Categorized by Age.

	Overall		Indoor		Outdoor- Street or Sidewalk		Outdoor- Not on Street or Sidewalk		Indoor/outdoor unclear		Missing	
	N = 1854909		N = 1596860		N = 129408		N = 23586		N = 53700		N = 51355	
**0–20**												
*Dead*	54	0.1%	42	0.1%	7	0.1%	2	0.0%	2	0.0%	1	0.1%
*Critical*	2205	2.6%	1592	2.4%	241	3.3%	164	3.0%	191	3.4%	17	1.8%
*Emergent*	18704	21.8%	14293	21.5%	1520	20.9%	1285	23.3%	1397	24.7%	209	22.1%
*Low*	64928	75.6%	50590	76.1%	5501	75.7%	4055	73.6%	4064	71.9%	718	76.0%
*Missing*	31314	26.7%	21585	24.5%	2588	26.3%	1515	21.6%	3700	39.6%	1926	67.1%
**22–30**												
*Dead*	44	0.1%	30	0.1%	10	0.1%	3	0.2%	0	0.0%	1	0.2%
*Critical*	1648	3.5%	1139	3.3%	295	3.5%	94	6.5%	103	4.9%	17	3.3%
*Emergent*	10996	23.4%	8009	23.3%	1834	21.8%	410	28.3%	605	28.8%	138	27.2%
*Low*	34217	72.9%	25262	73.4%	6265	74.5%	943	65.0%	1395	66.3%	352	69.3%
*Missing*	17177	26.8%	10651	23.6%	3051	26.6%	417	22.3%	1987	48.6%	1071	67.8%
**31–40**												
*Dead*	81	0.1%	51	0.1%	18	0.2%	5	0.3%	7	0.3%	0	0.0%
*Critical*	2224	3.7%	1685	3.7%	357	3.6%	65	4.1%	103	4.8%	14	2.2%
*Emergent*	14845	24.7%	11401	24.9%	2272	23.0%	415	26.3%	598	27.7%	159	25.0%
*Low*	42917	71.4%	32659	71.3%	7249	73.3%	1095	69.3%	1452	67.2%	462	72.8%
*Missing*	21942	26.8%	14314	23.8%	3636	26.9%	463	22.7%	2267	51.2%	1262	66.5%
**41–50**												
*Dead*	98	0.1%	77	0.1%	14	0.1%	5	0.3%	2	0.1%	0	0.0%
*Critical*	2866	3.7%	2250	3.7%	385	3.6%	91	5.8%	106	5.0%	34	4.3%
*Emergent*	19459	25.4%	15762	25.6%	2499	23.4%	417	26.5%	602	28.2%	179	22.7%
*Low*	54304	70.8%	43446	70.6%	7793	72.9%	1061	67.4%	1428	66.8%	576	73.0%
*Missing*	27486	26.4%	18781	23.4%	4030	27.4%	483	23.5%	2599	54.9%	1593	66.9%
**51–60**												
*Dead*	218	0.1%	194	0.2%	10	0.1%	6	0.3%	7	0.2%	1	0.1%
*Critical*	5277	3.6%	4399	3.6%	542	3.1%	143	6.4%	150	4.6%	43	2.8%
*Emergent*	37159	25.0%	31178	25.2%	4064	23.0%	639	28.7%	919	27.9%	359	23.3%
*Low*	105835	71.3%	87952	71.1%	13086	73.9%	1441	64.6%	2218	67.3%	1138	73.8%
*Missing*	52012	25.9%	37740	23.4%	6420	26.6%	633	22.1%	4181	55.9%	3038	66.3%
**61–65**												
*Dead*	183	0.2%	166	0.2%	7	0.1%	2	0.2%	4	0.2%	4	0.4%
*Critical*	3314	3.2%	2889	3.2%	253	2.7%	59	4.4%	81	4.2%	32	2.8%
*Emergent*	26404	25.5%	23022	25.7%	2220	23.8%	354	26.7%	528	27.3%	280	24.9%
*Low*	73459	71.1%	63559	70.9%	6855	73.4%	913	68.8%	1322	68.3%	810	71.9%
*Missing*	35026	25.3%	26870	23.1%	3312	26.2%	335	20.1%	2471	56.1%	2038	64.4%
**66–70**												
*Dead*	156	0.1%	146	0.1%	5	0.1%	2	0.2%	2	0.1%	1	0.1%
*Critical*	3733	3.2%	3268	3.1%	271	3.5%	78	6.3%	84	4.3%	32	2.5%
*Emergent*	29925	25.6%	26894	25.7%	1891	24.4%	308	24.9%	567	29.1%	265	20.8%
*Low*	82986	71.0%	74271	71.0%	5593	72.1%	847	68.6%	1298	66.5%	977	76.6%
*Missing*	39316	25.2%	31603	23.2%	2804	26.5%	362	22.7%	2190	52.9%	2357	64.9%
**71–75**												
*Dead*	154	0.1%	140	0.1%	7	0.1%	3	0.3%	3	0.2%	1	0.1%
*Critical*	3979	3.0%	3617	2.9%	198	2.9%	69	5.9%	68	3.9%	27	1.8%
*Emergent*	34609	25.7%	31780	25.7%	1693	24.9%	339	29.1%	505	28.8%	292	20.0%
*Low*	96078	71.3%	88117	71.3%	4888	72.0%	752	64.7%	1178	67.2%	1143	78.1%
*Missing*	46366	25.6%	38286	23.6%	2550	27.3%	318	21.5%	2094	54.4%	3118	68.1%
**76–80**												
*Dead*	174	0.1%	167	0.1%	5	0.1%	1	0.1%	0	0.0%	1	0.1%
*Critical*	4147	2.8%	3836	2.8%	170	2.9%	45	4.8%	71	4.5%	25	1.5%
*Emergent*	37589	25.6%	34923	25.6%	1637	27.5%	265	28.1%	448	28.2%	316	18.9%
*Low*	104879	71.4%	97704	71.5%	4143	69.6%	633	67.1%	1071	67.4%	1328	79.5%
*Missing*	50954	25.8%	42686	23.8%	2231	27.3%	255	21.3%	1892	54.3%	3890	70.0%
*Dead*	174	0.1%	167	0.1%	5	0.1%	1	0.1%	0	0.0%	1	0.1%
**81–85**												
*Dead*	149	0.1%	141	0.1%	5	0.1%	0	0.0%	1	0.1%	2	0.1%
*Critical*	4144	2.6%	3884	2.6%	138	2.8%	34	5.2%	58	4.4%	30	1.6%
*Emergent*	39227	24.9%	37019	24.9%	1318	26.8%	166	25.6%	330	25.2%	394	20.6%
*Low*	114226	72.4%	107913	72.4%	3459	70.3%	448	69.1%	918	70.2%	1488	77.7%
*Missing*	55089	25.9%	46615	23.8%	1873	27.6%	173	21.1%	1851	58.6%	4577	70.5%
**86–90**												
*Dead*	142	0.1%	139	0.1%	2	0.1%	0	0.0%	0	0.0%	1	0.1%
*Critical*	3658	2.4%	3491	2.4%	102	3.0%	23	5.4%	21	2.2%	21	1.1%
*Emergent*	37176	24.3%	35490	24.3%	915	27.3%	136	32.1%	274	28.8%	361	18.5%
*Low*	111970	73.2%	107146	73.3%	2333	69.6%	265	62.5%	656	69.0%	1570	80.4%
*Missing*	53358	25.9%	45313	23.7%	1270	27.5%	93	18.0%	1515	61.4%	5167	72.6%
**91+**												
*Dead*	120	0.1%	117	0.1%	1	0.1%	0	0.0%	0	0.0%	2	0.1%
*Critical*	3084	2.3%	2956	2.3%	54	3.1%	18	7.6%	23	4.0%	33	1.7%
*Emergent*	31846	23.7%	30788	23.7%	487	27.5%	72	30.4%	146	25.7%	353	18.4%
*Low*	99137	73.9%	95831	73.9%	1227	69.4%	147	62.0%	399	70.2%	1533	79.8%
*Missing*	46823	25.9%	39717	23.4%	724	29.0%	53	18.3%	1214	68.1%	5115	72.7%
Missing Age	13319	0.7%	11274	0.7%	1080	0.8%	168	0.7%	334	0.6%	463	0.9%

**Table 5:** Descriptive Statistics for EMS Encounters for Reported Location of Fall Injuries by Need for Triage to a Trauma Center, Categorized by Age.^1^

	Overall		Indoor		Outdoor- Street or Sidewalk		Outdoor- Not on Street or Sidewalk		Indoor/outdoor unclear		Missing	
	N = 1854909		N = 1596860		N = 129408		N = 23586		N = 53700		N = 51355	
**0–20**												
*Need for transport to Trauma Center*	6209	7.0%	4856	7.3%	609	7.8%	225	4.3%	389	5.1%	130	6.7%
*Does not*	82721	93.0%	61452	92.7%	7209	92.2%	5026	95.7%	7230	94.9%	1804	93.3%
*Missing*	28275	24.1%	21794	24.7%	2039	20.7%	1770	25.2%	1735	18.5%	937	32.6%
**22–30**												
*Need for transport to Trauma Center*	3229	5.8%	2166	5.5%	722	7.5%	76	5.0%	201	5.5%	64	5.1%
*Does not*	52521	94.2%	37470	94.5%	8953	92.5%	1453	95.0%	3459	94.5%	1186	94.9%
*Missing*	8332	13.0%	5455	12.1%	1780	15.5%	338	18.1%	430	10.5%	329	20.8%
**31–40**												
*Need for transport to Trauma Center*	4417	6.2%	3152	6.0%	908	7.9%	82	4.8%	205	5.1%	70	4.7%
*Does not*	67245	93.8%	49813	94.0%	10596	92.1%	1637	95.2%	3781	94.9%	1418	95.3%
*Missing*	10347	12.6%	7145	11.9%	2028	15.0%	324	15.9%	441	10.0%	409	21.6%
**41–50**												
*Need for transport to Trauma Center*	5493	6.0%	4167	5.9%	886	7.0%	82	4.7%	269	6.3%	89	4.8%
*Does not*	86218	94.0%	67011	94.1%	11781	93.0%	1646	95.3%	4028	93.7%	1752	95.2%
*Missing*	12502	12.0%	9138	11.4%	2054	14.0%	329	16.0%	440	9.3%	541	22.7%
**51–60**												
*Need for transport to Trauma Center*	10231	5.8%	8262	5.8%	1305	6.2%	114	4.8%	372	5.4%	178	5.0%
*Does not*	166488	94.2%	134674	94.2%	19704	93.8%	2258	95.2%	6464	94.6%	3388	95.0%
*Missing*	23782	11.9%	18527	11.5%	3113	12.9%	490	17.1%	639	8.5%	1013	22.1%
**61–65**												
*Need for transport to Trauma Center*	6664	5.5%	5710	5.5%	573	5.2%	60	4.3%	208	5.1%	113	4.6%
*Does not*	115516	94.5%	97482	94.5%	10496	94.8%	1349	95.7%	3853	94.9%	2336	95.4%
*Missing*	16206	11.7%	13314	11.4%	1578	12.5%	254	15.3%	345	7.8%	715	22.6%
**66–70**												
*Need for transport to Trauma Center*	7073	5.1%	6303	5.2%	387	4.2%	58	4.3%	177	4.6%	148	5.3%
*Does not*	130439	94.9%	114077	94.8%	8781	95.8%	1300	95.7%	3633	95.4%	2648	94.7%
*Missing*	18604	11.9%	15802	11.6%	1396	13.2%	239	15.0%	331	8.0%	836	23.0%
**71–75**												
*Need for transport to Trauma Center*	7729	4.8%	7089	5.0%	308	3.8%	50	3.9%	145	4.1%	137	4.1%
*Does not*	151707	95.2%	135990	95.0%	7862	96.2%	1241	96.1%	3387	95.9%	3227	95.9%
*Missing*	21750	12.0%	18861	11.6%	1166	12.5%	190	12.8%	316	8.2%	1217	26.6%
**76–80**												
*Need for transport to Trauma Center*	8219	4.7%	7617	4.8%	262	3.7%	32	3.2%	135	4.3%	173	4.2%
*Does not*	165532	95.3%	150722	95.2%	6884	96.3%	983	96.8%	3040	95.7%	3903	95.8%
*Missing*	23992	12.1%	20977	11.7%	1040	12.7%	184	15.3%	307	8.8%	1484	26.7%
**81–85**												
*Need for transport to Trauma Center*	8200	4.4%	7723	4.4%	159	2.7%	23	3.3%	105	3.6%	190	3.9%
*Does not*	179791	95.6%	165930	95.6%	5765	97.3%	676	96.7%	2795	96.4%	4625	96.1%
*Missing*	24844	11.7%	21919	11.2%	869	12.8%	122	14.9%	258	8.2%	1676	25.8%
**86–90**												
*Need for transport to Trauma Center*	7907	4.3%	7500	4.4%	127	3.1%	20	4.7%	90	4.0%	170	3.3%
*Does not*	174027	95.7%	162504	95.6%	3933	96.9%	410	95.3%	2179	96.0%	5001	96.7%
*Missing*	24370	11.8%	21575	11.3%	562	12.2%	87	16.8%	197	8.0%	1949	27.4%
**91+**												
*Need for transport to Trauma Center*	7095	4.4%	6707	4.5%	95	4.4%	15	6.3%	72	4.3%	206	4.0%
*Does not*	152599	95.6%	143729	95.5%	2082	95.6%	222	93.7%	1602	95.7%	4964	96.0%
*Missing*	21316	11.8%	18973	11.2%	316	12.7%	53	18.3%	108	6.1%	1866	26.5%
Missing Age	13319	0.7%	11274	0.7%	1080	0.8%	168	0.7%	334	0.6%	463	0.9%

1 T-RTS, Revised Trauma Score for Triage

## Abbreviations

BRFSS: Behavioral Risk Factor Surveillance System
EMS: Emergency Medical Services
WISQARS: Web-Based Injury Statistics Query and Reporting System
ICD 10: International Classification of Diseases 10th Revision
NHIS: National Health Interview Survey
NEMSIS: National Emergency Medical Services Information System
ISS: Injury Severity Score
GCS: Glasgow Coma Scale
T-RTS: Revised Trauma Score for Triage
ED: Emergency Departments
